# *In vitro* cytotoxicity of *Withania somnifera* (L.) roots and fruits on oral squamous cell carcinoma cell lines: a study supported by flow cytometry, spectral, and computational investigations

**DOI:** 10.3389/fphar.2024.1325272

**Published:** 2024-01-18

**Authors:** Ahmed Abdullah Al Awadh, Hiroshi Sakagami, Shigeru Amano, Ahmed M. Sayed, Mohamed E. Abouelela, Abdulaziz Hassan Alhasaniah, Nayef Aldabaan, Mohamed S. Refaey, Reda A. Abdelhamid, Heba M. A. Khalil, Dalia I. Hamdan, El-Shaymaa Abdel-Sattar, Mohamed A. A. Orabi

**Affiliations:** ^1^ Department of Clinical Laboratory Sciences, College of Applied Medical Sciences, Najran University, Najran, Saudi Arabia; ^2^ Meikai University Research Institute of Odontology (M-RIO), Saitama, Japan; ^3^ Department of Pharmacognosy, Faculty of Pharmacy, Nahda University, Beni-Suef, Egypt; ^4^ Department of Pharmacognosy, Collage of Pharmacy, Almaaqal University, Basra, Iraq; ^5^ Pharmacognosy Department, Faculty of Pharmacy (Boys), Al-Azhar University, Cairo, Egypt; ^6^ Department of Pharmacology, College of Pharmacy, Najran University, Najran, Saudi Arabia; ^7^ Department of Pharmacognosy, Faculty of Pharmacy, University of Sadat City, Sadat City, Menoufiya, Egypt; ^8^ Department of Pharmacognosy, Faculty of Pharmacy, Al-Azhar University, Assiut-Branch, Assiut, Egypt; ^9^ Department of Veterinary Hygiene and Management, Faculty of Veterinary Medicine, Cairo University, Giza, Egypt; ^10^ Department of Pharmacognosy and Natural Products, Faculty of Pharmacy, Menoufia University, Shibin Elkom, Egypt; ^11^ Department of Medical Microbiology and Immunology, Faculty of Pharmacy, South Valley University, Qena, Egypt; ^12^ Department of Pharmacognosy, College of Pharmacy, Najran University, Najran, Saudi Arabia

**Keywords:** *Withania somnifera*, oral cancer, flow cytometry, UHPLC MS/MS, CDK2, BRD3, molecular docking, molecular dynamics

## Abstract

Oral cancer is a severe health problem that accounts for an alarmingly high number of fatalities worldwide. *Withania somnifera* (L.) Dunal has been extensively studied against various tumor cell lines from different body organs, rarely from the oral cavity. We thus investigated the cytotoxicity of *W. somnifera* fruits (W-F) and roots (W-R) hydromethanolic extracts and their chromatographic fractions against oral squamous cell carcinoma (OSCC) cell lines [Ca9-22 (derived from gingiva), HSC-2, HSC-3, and HSC-4 (derived from tongue)] and three normal oral mesenchymal cells [human gingival fibroblast (HGF), human periodontal ligament fibroblast (HPLF), and human pulp cells (HPC)] in comparison to standard drugs. The root polar ethyl acetate (W-R EtOAc) and butanol (W-R BuOH) fractions exhibited the strongest cytotoxicity against the Ca9-22 cell line (CC_50_ = 51.8 and 40.1 μg/mL, respectively), which is relatively the same effect as 5-FU at CC_50_ = 69.4 μM and melphalan at CC_50_ = 36.3 μM on the same cancer cell line. Flow cytometric analysis revealed changes in morphology as well as in the cell cycle profile of the W-R EtOAc and W-R BuOH-treated oral cancer Ca9-22 cells compared to the untreated control. The W-R EtOAc (125 μg/mL) exerted morphological changes and induced subG_1_ accumulation, suggesting apoptotic cell death. A UHPLC MS/MS analysis of the extract enabled the identification of 26 compounds, mainly alkaloids, withanolides, withanosides, and flavonoids. Pharmacophore-based inverse virtual screening proposed that BRD3 and CDK2 are the cancer-relevant targets for the annotated withanolides D (**18**) and O (**12**), and the flavonoid kaempferol (**11**). Molecular modeling studies highlighted the BRD3 and CDK2 as the most probable oncogenic targets of anticancer activity of these molecules. These findings highlight *W. somnifera*’s potential as an affordable source of therapeutic agents for a range of oral malignancies.

## 1 Introduction

As the second main cause of death among non-communicable diseases, cancer-related deaths are increasing at an alarming rate (Lee et al., 2016). Cancer can originate anywhere in the human body areas including, the oral cavity, lung, breast, liver, prostate, colon, kidney, ovary, etc. (Ferlay et al., 2021). There are about 500,000 new incidences of oral cancer reported annually worldwide, and rising trends have been reported in many countries, particularly among tobacco smokers. According to the American Cancer Society’s update, there will be around 54,000 new instances of oral cavity and oropharyngeal cancer in the United States in 2022, along with 11,230 fatalities (Siegel et al., 2022). A similar increase was reported in Saudi Arabia; the Saudi Cancer Registry found 3,184 incidences of oral cancer during the period 1994–2015 (Alshehri, 2020). The most typical locations for oral cavity and oropharyngeal malignancies include the tongue, tonsils, oropharynx, gums, and mouth floor, in addition to the roof of the mouth and small salivary glands (Cai et al., 2023). There is a direct link between oral cancers and age, as well as between oral cancer and smoking, especially shisha (smoking through a water pipe), which is very popular in the Middle East (Etemadi et al., 2017). Generally, the possibility of developing cancers in the oral cavity and oropharyngeal tissues is about 1/60 for men and 1/140 for women (Gupta et al., 2022).

Despite the availability of anticarcinogenic drugs, the vast bulk of them are cost-prohibitive and come with several side effects. As a result, enticing natural, cost-effective drugs, with the least side effects are pressingly aimed.

The most integrated development of anticancer therapy is thought to be plant-based immunomodulatory agents (Ali et al., 2021). *Withania somnifera* (L.) Dunal, belonging to the family Solanaceae, is an important medicinal herb with well-established immunostimulatory activity (Davis and Kuttan, 2000). Macromorphologically, *W. somnifera* is an erect, thick, hairy, greyish-tomentose herb or under-shrub that can reach a height of 1.5 m. Its primary stem bears simple, dull green, glabrous, elliptic, petiolated, whole, opposite leaves, and bell-shaped flowers on upright, tomentose branches. Its fruits are shaped like green berries and mature to orange-red colour. Its taproot system is pale yellow in hue (Macharia et al., 2023). It is famous as Indian Ginseng, Ashwagandha, Ajagandha, Kanaje Hindi, Winter Cherry, and Samm Al Ferakh, and has various medicinal uses in the ancient Indian medical system (Ayurveda) (Singh et al., 2021). It was originally prescribed to treat problems with infertility, but afterward, it is frequently used to cure anxiety, increase vital fluid and lymph production, fight aging, and increase vigour and muscle strength (Chaurasiya et al., 2009; MR et al., 2010; John, 2014).

*W. somnifera* has attracted large attention regarding its anticancer properties. *W. somnifera* extracts and purified compounds, mainly withanolides, alkaloids, and phenolics, have been studied for their cytotoxic effects against a variety of cancer cell lines [*viz*. A549, and H1299, and NCI–H460 (non-small cell lung cancer, NSCLC), SW480, HCT-15, HCT-116, and RKO (human colorectal cancer), SF-268 (brain cancer), and MCF-7 (breast cancer), AGS (human gastric cancer), HL-60 (human promyelocytic leukemia), MDA-MB-231 (estrogen-independent mammary cancer cell line), PC-3 and DU-145 (prostate cancer), IMR-32 (neuroblastoma), K562 (human erythroleukemia), MOLT-4 (lymphoid leukemia), SUM 159 and T47D (mammary cancer), A2780 cell line (ovarian cancer), A375 (human malignant melanoma; skin tumor), Huh 7 and MHCC97H (liver), and C6 (rat glioma), and YKG1 (human glioma)] (Rai et al., 2016; Singh et al., 2021).

The promising effects of *W. somnifera* against cancer cell lines from almost all body organs, rarely from the oral cavity, coupled with our interest in finding anti-oral cancer drug candidates (Orabi et al., 2021), endorsed us to screen *W. somnifera* roots and fruits extracts to exploit it against oral cancers.

In this study, the specific cytotoxicity of ten different extracts and fractions from roots and fruits of *W. somnifera* was determined against human oral squamous cell carcinoma (HOSCC) cell lines [Ca9-22 (human gingival squamous carcinoma cell line), HSC-2, HSC-3, and HSC-4 (human squamous carcinoma, derived from tongue)], and three normal oral mesenchymal cells [human gingival fibroblast (HGF), human periodontal ligament fibroblast (HPLF), and human pulp cells (HPC)], and the results were compared with 5-fluorouracil (5-FU), doxorubicin, and melphalan standard chemotherapeutics. Further, flow cytometric analysis was performed to recognize the possible cytotoxic mechanism. The *Withania* active components were then explored using two-stage UHPLC-ESI MS/MS mass spectrometry. To figure out the appropriate configurations of biomolecular ligands and to gauge how well a ligand interacts with the protein, molecular docking of the identified *W. somnifera* metabolites with cyclin-dependent kinase 2 (CDK2) and bromodomain-containing protein 3 (BRD3) proteins was performed. The compounds with the best binding affinity were further refined by molecular dynamics (MD) simulations.

## 2 Materials and methods

### 2.1 General experimental procedures

Following informed consent from the patient at Meikai University Hospital, the 12-year-old girl had her first premolar tooth pulled from her lower jaw following Intramural Ethic Committee guidelines (No. A0808). The lower jaw first premolar tooth was used for establishing the HGF, HPLF, and HPC cells, and HOSCC cell lines [Ca9-22 (Catalog number: RCB-1976), HSC-2 (RCB-1945), HSC-3 (RCB-1975), and HSC-4 (RCB-1902)], purchased from Riken Cell Bank, Tsukuba, Japan. The Dulbecco’s modified Eagle’s medium (Catalog #: 30-2002), kanamycin (CAS #: 133-92-6), dimethyl sulfoxide (DMSO) (CAS #:67-68-5), and 3-(4,5-dimethythiazol-2yl)-2,5-diphenyltetrazolium bromide (MTT) (CAS #: 298-93-1), and doxorubicin (CAS #: 25316-40-9) were purchased from FUJIFILM Wako Chem, Osaka, Japan. 5-fluorouracil (5-FU) (CAS #: 51-21-8) was purchased from Kyowa, Tokyo, Japan. Fetal bovine serum (FBS) (Catalog #: 16000044) and melphalan (CAS #: 148-82-3) were obtained from Sigma-Aldrich (St. Louis, MO, United States). The 100 mm dishes and 96-well plates used for the culture were purchased from True Line (Haryana, India) and TPP (Techno Plastic Products AG, Trasadingen, Switzerland), respectively.

### 2.2 Plant material

The roots and fruits of *W. somnifera* were harvested (in October 2019) from plants rife in the vicinity of the Colleges of Pharmacy and Applied Medical Sciences at Najran University (geographic location, 17.633418, 44.5383887, Saudi Arabia. The plant was authenticated by Prof. Omer H. Mohamed Ibrahim, Arid Land Agriculture Department, Faculty of Meteorology, Environmental, and Arid Land Agriculture, King Abdul-Aziz University, SA. The roots were cleaned from the remaining clay, cut into small pieces, and dried in the shade. The fruits were deprived of any floral parts and dried in shade. Voucher specimens [Root (AshR-10/018) and fruit (AshF-10/018)] were kept at the Department of Pharmacognosy, College of Pharmacy, Najran University, SA.

### 2.3 Extraction and fractionation

*W. somnifera* roots and fruits (each 200 g dry powder) were separately extracted by MeOH–H_2_O (8:2, *v/v*, 4 × 1.5 L) using homogenizer. The extracts were vacuum dried at 40°C by rotary evaporators and yielded total extracts [W-R total (37.4 g, 19%) and W-F total (44.96 g, 22%, *w/w*)], respectively. Samples (6 and 4g, respectively) of both extracts were kept for biological assessments. The remains of the total extracts were chromatographed on silica gel (70–230 mesh) [(25 × 7 cm, i. d.] with *n*-hexane, EtOAc, *n*–butanol, and MeOH (3L each), successively. The different eluates were vacuum dried at 40°C and afforded the corresponding dry sub-extracts [root (0.29, 0.58, 1.79, and 28.7 g), fruit (8.43, 1.94, 6.4, and 24.3 g)], respectively. The different extracts and sub-extracts/fractions were kept in sample vials for spectro-chromatographic and biological investigations.

### 2.4 Cytotoxicity assay

The cancer cell lines (HSC-2, HSC-3, HSC-4), as well as the normal cells (HGF, HPLF, and HPC), were cultured at 37°C in a humidified 5% CO_2_ incubator in DMEM medium enriched with streptomycin sulphate (100 μg/mL), 10% heat-inactivated FBS, and penicillin G (100 units/mL). For cytotoxicity testing, cells were then garnered using 0.25% trypsin–0.025% sodium edetate in a phosphate-buffered saline lacking Ca^2+^/Mg^2+^ [PBS (−)]. The cells were cultured in 96-well microplates at a cell count of 3 ×10^3^ cells/100 μL. After 2 days, the exhausted medium was replaced with fresh medium containing the different concentrations of sample to be tested, in triplicate. The initial sample concentration was set at 5 mg/mL in DMSO. The first concentration examined was thus 500 μg/mL, which was then successively diluted 2-fold. In the wells assigned for control, equal volumes of the DMSO were added to the cells, where the toxic effects of DMSO could be subtracted. The cell growth in the incubator was continued for another 2 days. To determine the cell viability, the colorimetric MTT method was employed as described in our preceding article (Orabi et al., 2021). The concentration of cytotoxicity 50% (CC_50_) was determined from the dose-response curve. The mean CC_50_ value for each type of cell was calculated from triplicate assays (Kantoh et al., 2010). The changes in cell morphology were detected using light microscopy (EVOSfl; ThermoFisher Scientific, Waltham, MA, United States).

### 2.5 Calculation of the TS index

The following equation was used to calculate tumor specificity (TS): TS = average CC_50_ toward the normal cells (HGF + HPLF + HPC)/average CC_50_ toward the tumor cell lines (Ca9-22 + HSC-2 + HSC-3 + HSC-4), as indicated by D/B (Table 1). To compare the sensitivity of the cancer cells (Ca9-22) with that of the normal cells (HGF), being generated from the same tissue, the equation TS = CC_50_ against HGF/CC_50_ against Ca9-22 was used (see C/A in Table 1).

**TABLE 1:** Quantification of tumor selectivity (TS) and potency-selectivity expression (PSE) values of *W. somnifera* fractions (W-R EtOAc and W-R BuOH) and the standard cytotoxic (doxorubicin, melphalan) and cytostatic drugs (5-FU) against OSCC cell lines and normal oral cells.

Extracts and fractions		CC_50_ (ug/mL or μM)												
		Human oral squamous cell carcinoma cell lines					Human normal oral cells							
		Ca9-22	HSC-2	HSC-3	HSC-4	Mean	HGF	HPLF	HPC	Mean	TS^a^		PSE^b^	
		(A)				(B)	(C)			(D)	D/B	C/A	100D/B^2^	100C/A^2^
W-F Total	(μg/mL)	>500	>500	>500	>500	>500	>500	>500	>500	>500	1.0	1.0	0.2	0.2
W-F Hex	(μg/mL)	>500	>500	>500	>500	>500	>500	>500	>500	>500	1.0	1.0	0.2	0.2
W-F EtOAc	(μg/mL)	408	>500	439	464	>453	>500	>500	>500	>500	1.1	1.2	0.2	0.3
W-F BuOH	(μg/mL)	>500	>500	>500	>500	>500	>500	>500	>500	>500	1.0	1.0	0.2	0.2
W-F MeOH	(μg/mL)	>500	>500	>500	>500	>500	>500	>500	>500	>500	1.0	1.0	0.2	0.2
W-R Total	(μg/mL)	466	>500	>500	>500	>491	>500	>500	>500	>500	1.0	1.1	0.2	0.2
W-R Hex	(μg/mL)	>500	>500	>500	>500	>500	>500	>500	>500	>500	1.0	1.0	0.2	0.2
W-R EtOAc	(μg/mL)	51.8	88.8	98.7	76.1	78.9	186	167	187	180	2.3	3.6	2.9	6.9
W-R BuOH	(μg/mL)	40.1	96.6	77.1	88.1	75.5	295	>500	335	>376	5.0	7.3	6.6	18.3
W.R MeOH	(μg/mL)	>500	>500	>500	>500	>500	>500	>500	>500	>500	1.0	1.0	0.2	0.2
Doxorubicin	(μM)	0.18	0.09	0.16	<0.078	0.13	>10	>10	>10	>10	78.5	54.9	61,611.4	30,189.6
5-FU	(μM)	69.4	292	>1,000	182	386	>1,000	>1,000	>1,000	>1,000	2.6	14.4	0.7	20.8
Melphalan	(μM)	36.3	11.0	14.1	9.9	17.8	180	>200	159	180	10.1	5.0	56.6	13.7

^a^ TS, value was calculated using the following equation; TS, mean CC_50_ against three normal human oral mesenchymal cells (HGF + HPLF + HPC)/mean CC_50_ against four OSCC, cell lines (Ca9-22 + HSC-2 + HSC-3 + HSC-4), as shown by D/B. The relative sensitivity of Ca9-22 and HGF, cells is shown by C/A).

^b^ PSE, value was calculated using the following equation; PSE, 100 × TS/CC_50_ (tumor cells); (100 × D/B^2^) (three normal oral cells vs. four OSCC, cell lines) and 100 × C/A^2^ (HGF, vs. Ca9-22).

### 2.6 Calculation of the PSE index

Elevated values of TS and PSE (potency-selectivity expression) parameters indicate treatment of cancer patients at minimum damage to the normal cells. The PSE for the three normal oral cells vs. the four cancer cell lines was computed from the equation: PSE = 100 × TS/CC_50_ (tumor cells) (100 × D/B^2^). The PSE for the HGF vs. Ca9-22 was calculated from 100 × C/A^2^ (Table 1) (Takao et al., 2020).

### 2.7 Cell cycle analysis

Ca9-22 cells (3 × 10^4^/mL, 10 mL) were added to a 10-cm dish and allowed to fully attach to the dish over the course of 48 h of incubation. The culture medium was substituted with 10 mL of either new culture medium without (control) or with actinomycin D (1 μM) (positive control of apoptosis inducer), EtOAc extract (31.3, 62.5, 125, or 250 μg/mL), or BuOH extract (31.3, 62.5, 125, or 250 μg/mL). Following a 20-h incubation period, connected cells were separated by treating them with 0.25% trypsin-EDTA, and loosely bound and unattached cells were collected by centrifugation. These cells were mixed and then washed once with PBS (−). Both cell groups were fixed for 1 h on ice with 1% paraformaldehyde. Fixed cells were then washed twice with PBS (−) and treated with 400 μL of 200 μg/mL RNase A (which was preheated for 10 min at 100°C to inactivate DNase) to degrade RNA. The cells were then washed twice with PBS (−) and stained for 15 min with 0.01% propidium iodide (PI) in the presence of 0.01% NP-40 in PBS (−) to prevent cell aggregation. After filtering through Falcon^®^ cell strainers (nylon mesh, pore size: 40 μm) (Corning, NY, United States) to remove aggregated cells, PI-stained cells were then subjected to cell sorting using (SH800 Series; SONY Imaging Products and Solutions Inc., Kanagawa, Japan), and finally analyzed with the Cell Sorter Software version 2.1.2. (SONY Imaging Products and Solutions Inc., Kanagawa, Japan), as explained before (Takao et al., 2020).

### 2.8 Metabolite analysis using UHPLC-MS/MS

For LC-MS/MS analysis of the extract, a Shimadzu LC-10 HPLC, equipped with a Grace Vydac Everest Narrow bore C-18 column [internal diameter (100 mm × 2.1 mm) and particle size 300 Å), was utilized. An LTQ Linear Ion Trap MS (Thermo Finnigan, San Jose, CA), with a mass range of 100–2,000 *m/z*, was utilized. A sample size of 2 µL was auto-injected. A gradient elution pattern (continued for 15 min) was employed using gradients of 5% CH_3_CN and 0.05% HCOOH, until 95% CH_3_CN 0.05% HCOOH. For data analysis and interpretation, the software MSDIAL ver.5.1.230912 and MZmine 3 were utilized. The files of the raw data were then converted to mzXML format using MSConvert from the ProteoWizard suite (Al Mousa et al., 2022).

### 2.9 Computational investigation

#### 2.9.1 Virtual target identification

The putative target characterization of retinol was achieved via Pharmacophore-based Virtual screening using PharmMapper (Wang et al., 2017). This platform assigns a score to each molecule in the Protein Data Bank (PDB) that best fits a pharmacophore model that has been extracted and stored as a library of ligand datasets in mol2 format. To identify where a new molecule fits on the scale of all the pharmacophore scores, its fit score for each pharmacophore is determined, and each fit score is compared to the fit score matrix. The pure fit score that results from this procedure carries considerably more weight and assurance. The query structure was submitted to the platform in the PDB format, and the retrieved results were exported as an Excel sheet arranging the resulted protein targets according to their fit scores.

#### 2.9.2 Docking study

The docking investigation was carried out on the crystal structures of the CDK2 and BRD3 protein (PDB ID: 6GUB, and 7S3B) using AutoDock Vina (Huey et al., 2012). To determine the binding site and the docking grid-box in each protein structure, the respective co-crystallized ligand (Flavopiridol and physachinoloide C) was employed. The grid box coordinates were x = −30.626, y = −0.508, z = 33.564, and x = 34.364, y = −18.457, z = 5.761, respectively. The root-mean-square-deviation (RMSD) criterion for ligand-to-binding site shape matching was established at 2.0 Å. The Charmm force field (v.1.02) with a distance-dependent dielectric and a non-bonded cutoff distance of 10.0 Å was used to calculate the interaction energies. Next, an energy grid extending from the binding site was set at 5.0 Å. Energy minimization of the investigated compounds was achieved inside the designated binding pocket. Pymol software was used to edit and visualize the produced binding postures (Yuan et al., 2017).

#### 2.9.3 Molecular dynamics simulation (MDS)

The MDS analysis was performed by the NAMD 3.0.0 software (Phillips et al., 2005; Ribeiro et al., 2018), and the Charmm-36 force field was applied. The amino acids protonation states were adjusted (pH = 7.4), and the co-crystallized H_2_O molecules were removed. Using the QwikMD toolset of the VMD program, the protein molecules were verified for any lost hydrogen atoms (Humphrey et al., 1996; Ribeiro et al., 2018). The total molecule was then dipped in an orthorhombic TIP3P H_2_O box holding 0.15 M Na^+^ and Cl^−^ ions and a solvent buffer of 20 Å. The system’s energy was minimized and equilibrated for 5 ns. The VMD plugin Force Field Toolkit (ffTK) was used to calculate the properties and topologies of the ligands. The files of the parameters and topology were then located in VMD to rapidly read the protein-ligand complexes and perform the simulation phases.

#### 2.9.4 Binding free energy (BEE) calculations

To compute the BEE of the docked complex, the Molecular Mechanics Poisson-Boltzmann Surface Area rooted in the MMPBSA. py module of Assisted Model Building with Energy Refinement 2018 (AMBER18) was utilized (Miller et al., 2012). After processing 100 frames from the trajectories, the net energy of the system was calculated with the help of the Eq. 1:

$$\Delta G_{\text{Binding}}=\Delta G_{\text{Complex}}\;–\;\Delta G_{\text{Receptor}}\;–\;\Delta G_{\text{Inhibitor}}$$ (1)

Van der Waals energy, electrostatic energy, internal energy from molecular mechanics, and the polar contribution to solvation energy are among the several energy components that must be calculated for each of these concepts.

### 2.10 Statistical analysis

All analyses were carried out in triplicate to ensure robustness and reliability. The data are presented as mean ± standard deviation (SD). Graph Pad Prism 7 and Microsoft Excel 2010 were used for the statistical and graphical evaluations. For multiple comparisons, one-way analysis of variance (ANOVA) followed by Bonferroni’s *post hoc* test was performed (SPSS version 27.0). A value of *p* < 0.05 was considered to indicate statistically significant differences.

## 3 Results

### 3.1 Cytotoxic activity

**FIGURE 1:**
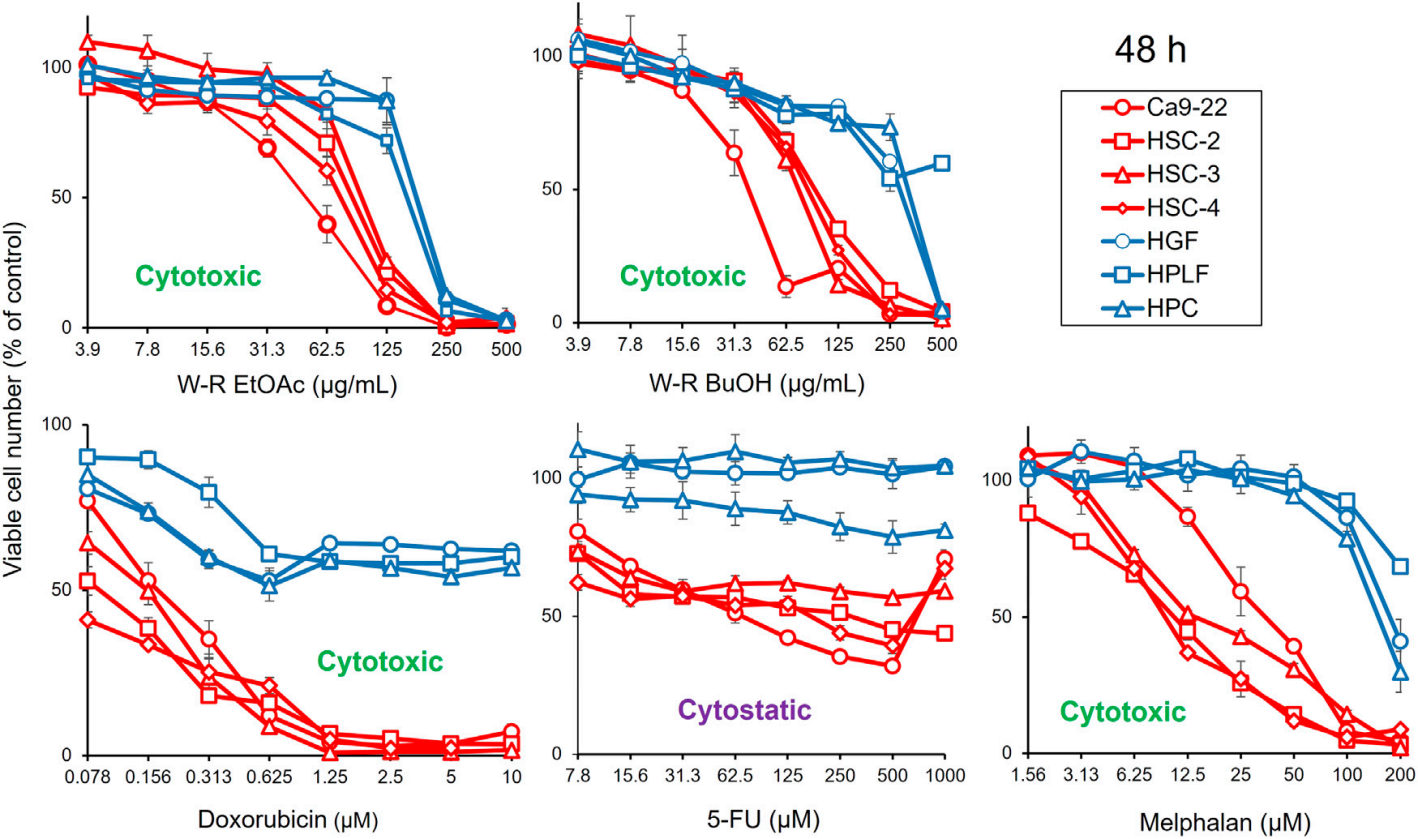
Dose-response curve of growth inhibition by *W. somnifera* fractions (W-R EtOAc and W-R BuOH), and the standard cytotoxic (doxorubicin, melphalan) and cytostatic (5-FU) drugs against OSCC cell lines (represented by red coloured curves) and normal oral cells (represented by blue coloured curves).

The results presented in Figure 1; Table 1 indicated the occurrence of the effective cytotoxic components in the root EtOAc (W-R EtOAc) and butanol (W-R BuOH) fractions. They exhibited strong cytotoxicity against the Ca9-22 cell line (CC_50_ = 51.8 and 40.1 μg/mL, respectively), which is relatively the same effect as 5-FU at CC_50_ = 69.4 μM and melphalan at CC_50_ = 36.3 μM on the same cancer cell line. Both the W-R EtOAc and W-R BuOH fractions exhibited moderate to low cytotoxicity CC_50_ = 76.1–98.7 μg/mL toward the other tumor cell lines, but very low or negligible cytotoxicity (CC_50_ = 167–>500 μg/mL) against the normal cells. The same fractions showed TS indices (TS = 2.3 and 5, respectively), calculated by dividing the mean CC_50_ toward the normal cells (HGF + HPLF + HPC) over the mean CC_50_ against the OSCC (Ca9-22 + HSC-2 + HSC-3 + HSC-4) cell lines (See D/B in Table 1), comparable to that of 5-FU and melphalan (TS = 2.6 and 10.1, respectively). The potency-selectivity expression (PSE), computed from the equation: PSE = 100 × TS/CC_50_ (tumor cells) (100 × D/B^2^) (three normal oral cells vs. four OSCC cell lines) and 100 × C/A^2^ (HGF vs. Ca9-22). As shown in Figure 1; Table 1, the W-R EtOAc and W-R BuOH exhibited PSE = 2.9 and 6.6, respectively) much higher than that of 5-FU (PSE = 0.7). The relative sensitivity of the gingival tissue-derived cells (Ca9-22 and HGF) was also compared (Table 1). The W-R EtOAc and W-R BuOH fractions showed TS = 3.6 and 7.3 and PSE = 6.9 and 18.3, respectively) comparable with 5-FU and melphalan (TS = 14.4 and 5.0 and PSE = 20.8 and 13.7, respectively).

### 3.2 Flow cytometry

**FIGURE 2:**
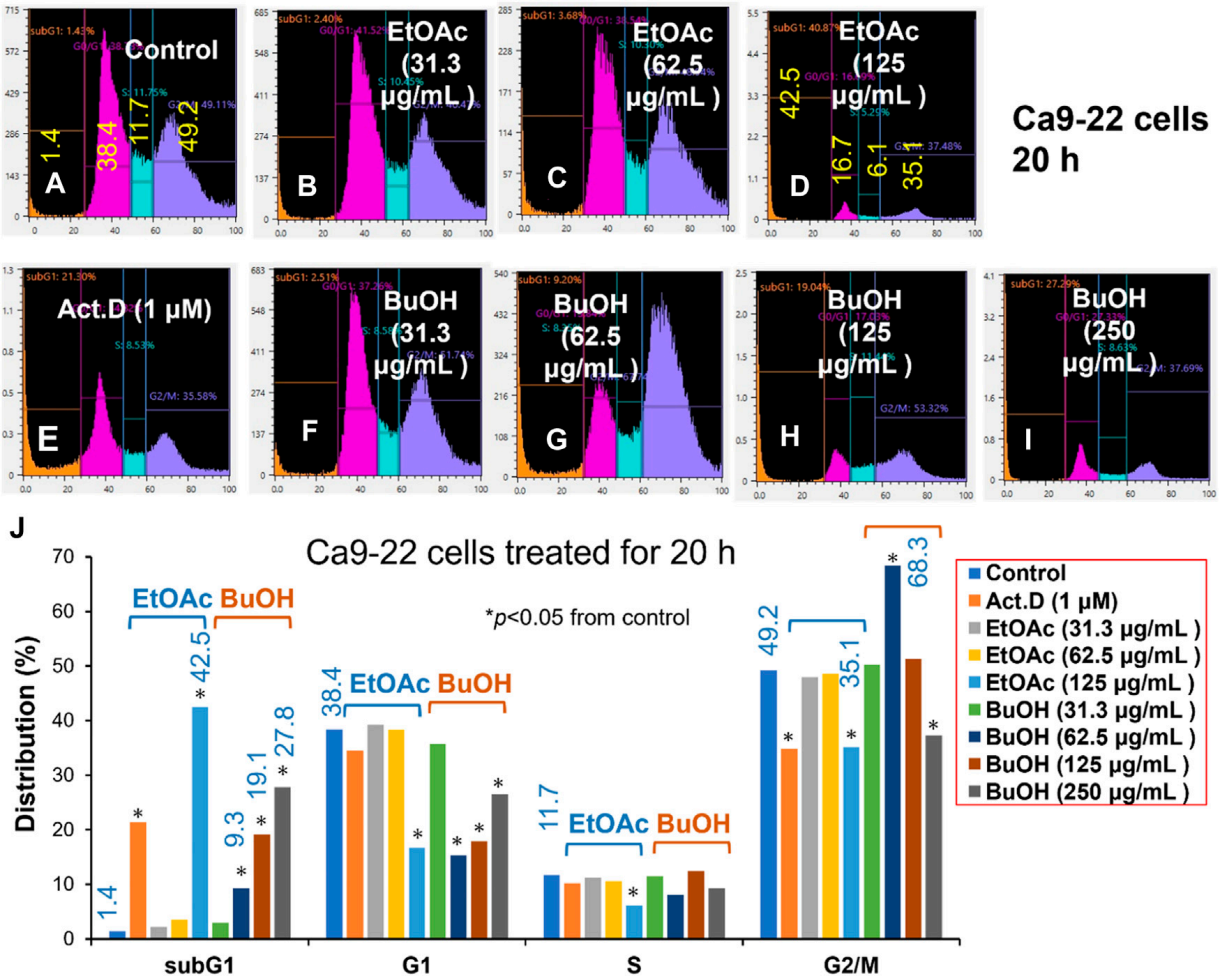
Cell cycle analysis of cytotoxicity induction by *W. somnifera* fractions (W-R EtOAc and W-R BuOH). Ca9-22 cells were incubated for 20 h with the indicated concentrations of test samples (W-R EtOAc and W-R BuOH) and subjected to a cell sorter. Upper panel: Representative cell cycle distribution pattern of the control **(A)** and cells treated with different concentrations of {W-R EtOAc [31.3 **(B)**, 62.5 **(C)**, and 125 **(D)** μg/mL], W-R BuOH [31.3 **(F)**, 62.5 **(G)**, 125 **(H)**, and 250 **(I)** μg/mL]}, and the standard Act. D (1 μM) **(E)**. Lower panel **(J)** Distribution into subG_1_, G_1_, S, and G_2_/M phases. Each value is represented as the mean ± S.D. of triplicate determinations. *p < 0.05 vs. control (Bonferroni’s post-test).

**FIGURE 3:**
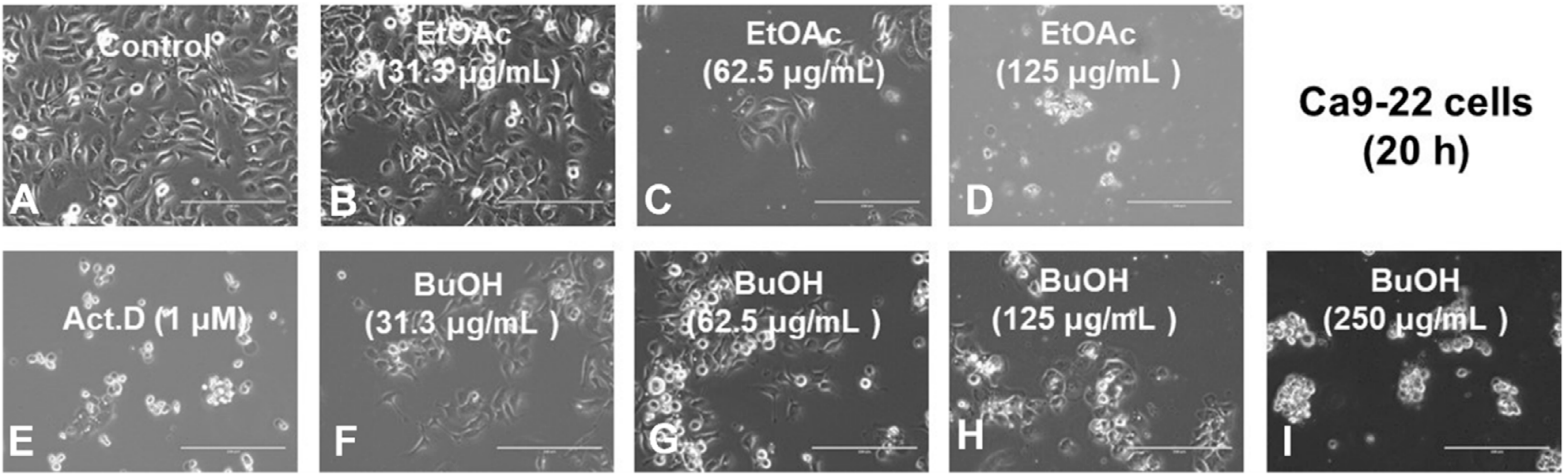
Morphological changes induced in Ca9-22 cells after 20 h incubation without a sample control, **(A)**, with the indicated concentrations of test samples {W-R EtOAc [31.3 **(B)**, 62.5 **(C)**, and 125 **(D)** μg/mL], W-R BuOH [31.3 **(F)**, 62.5 **(G)**, 125 **(H)**, and 250 **(I)** μg/mL], and the standard Act. D (1 μM) **(E)**}.

Since Ca9-22 cells were most sensitive to W-R EtOA and W-R BuOH among four OSCC cell lines (Table 1), their effects on the cell cycle and cell morphological changes in Ca9-22 cells were next investigated (Figures 2, 3), using actinomycin D (1 µM), positive control that induced cell shrinkage (a morphological hallmark of apoptosis) (Figure 3E) and a significant increase in the subG_1_ population (composed of DNA fragments) (Figure 2E).

The effective concentration range of W-R EtOAc was narrow. No significant variation in the cell cycle distribution was detected at 31.3 (Figure 2B) and 62.5 (Figure 2C) μg/mL of W-R EtOAc, but a significant variation in the cell cycle distribution was detected at 125 μg/mL (Figure 2D). The proportion of the subG_1_ population (composed of DNA fragments) (Figure 2D) was increased by 41.1% from the baseline (control, Figure 2A) (=42.5–1.4), whereas the proportions of G_1_, S, and G_2_/M phase cells were decreased by 21.7% (=16.7–38.4), 5.6% (=6.1–11.7), and G_2_/M 14.1% (=35.1–49.2), respectively (Figure 2J). Morphological observation demonstrated (Figures 2I, 3A) that cells became larger at the lower concentration range [31.3 and 62.5 μg/mL, Figures 3B, C, respectively)], whereas cells treated at a higher concentration 125 μg/mL were rather shrunken (Figure 3D), in parallel with significant increase in the subG_1_ population (Figure 2D).

In contrast, the effective concentration range of W-R BuOH was much wider, and the subG_1_ population increased significantly and dose-dependently from 1.4% (control) to 9.3, 19.1% and 27.8% at 62.5, 125, and 250 μg/mL (Figures 2G, H, I, respectively). Interestingly, the proportion of cells in the G_2_/M phase was significantly increased up to 19.1% (=68.3–49.2, *p* < 0.05), but began to decrease at the maximum concentration (125 μg/mL), possibly due to the shift to subG_1_ population (Figure 2). At this time, the cells were slightly larger at 62.6–125 μg/mL (Figures 3G, H). On the other hand, both W-R EtOAc and W-R BuOH did not increase, but rather reduced the distribution into G_1_ phase of cell cycle and did not cause apparent change in S phase cells. Based on these data and calculation, it was suggested that W-R EtOAc and W-R BuOH may first stimulate the accumulation of Ca9-22 cells in the G_2_/M phase having larger cell volume than other phases of the cell cycle, and then accumulate higher amount of subG_1_ population than actinomycin D.

### 3.3 Identification of *W. somnifera* metabolites

**FIGURE 4:**
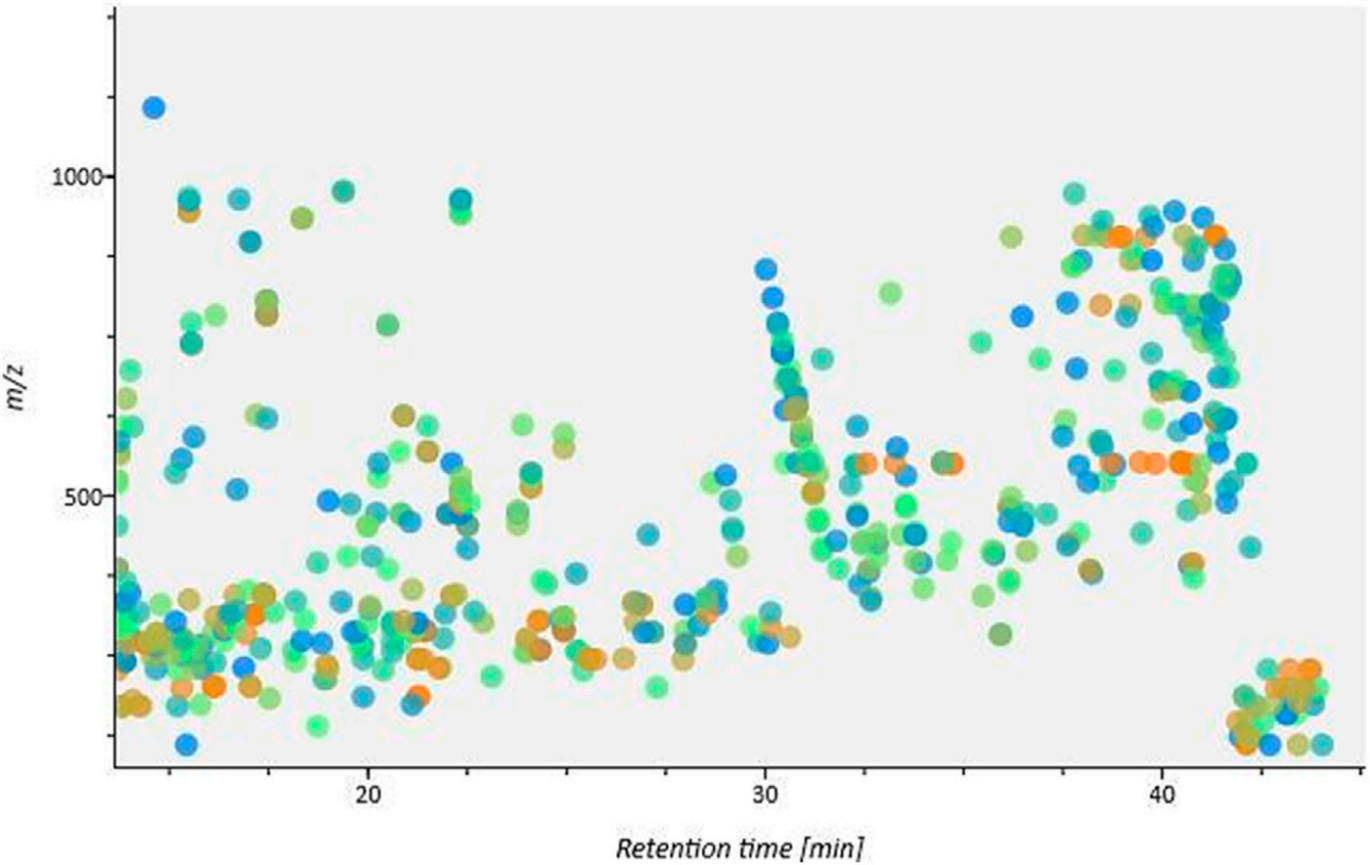
The heat map for detected peaks shows the distribution of *m/z* against retention time.

An analysis was carried out utilizing UHPLC-Q-TOF-MS/MS in the positive ion mode. The screening with MSDial revealed that each chromatographic peak frequently represents a variety of compounds (Figure 4). By checking for the molecular ion (m/z) value, the peaks were exactly located regardless of chromatographic settings or alterations to the instrumentation. Two-stage mass spectrometry enabled precise information about the elemental composition and characteristic fragment ions of each compound. Based on compound classes and structural information from online databases, the results were then compared to known compounds using calculated and reported masses (MS^1^), secondary masses (MS^2^), and specific fragmentation patterns. The analysis led to the tentative identification of twenty-six compounds, the majority of which belong to withanolides, withanosides along with other phytochemical classes like alkaloids, flavonoids, and steroids. Table 2 displays data for each identified chemical constituent’s retention time, chemical formula, molecular ion (MS^1^), and MS^2^ fragments. The compounds in the table are arranged according to their retention times.

**TABLE 2:** List of identified phytomolecules in *W. somnifera* extract.

No	Identified compound	Molecular formula	Retention time	Molecular weight	*m/z* (M + H)	MS^2^ fragments	Reference
1	Isopelletierine	C_8_H_15_NO	2.04	141.00	142.00	124, 114, 98	Singh et al. (2011)
2	Withasomnine	C_12_H_12_N_2_	13.49	184.10	185.18	183, 168, 158, 144, 141	Singh et al. (2011)
3	Daidzein	C_15_H_10_O_4_	13.59	254.06	255.26	237, 227, 223, 199	Jadhav et al. (2012)
4	Genistein	C_15_H_10_O_5_	14.16	270.05	271.30	253, 243, 225, 201, 197, 137	Jadhav et al. (2012)
5	Withaninsams-B	C_16_H_22_O_5_	16.38	294.15	295.20	277, 259, 237, 137, 123	Baek et al. (2019)
6	Withaninsams-A	C_16_H_22_O_5_	16.45	294.15	295.27	277, 259, 237, 137, 123	Baek et al. (2019)
7	Withanoside VI	C_40_H_62_O_15_	17.46	782.41	783.18	765, 621, 459, 441, 423	Matsuda et al. (2001)
8	Withanoside IV	C_40_H_62_O_15_	18.02	782.41	783.12	621, 459, 441, 423	Matsuda et al. (2001)
9	Withanoside II	C_40_H_62_O_16_	18.48	798.40	799.04	781, 771, 751, 683, 637, 485, 473	Matsuda et al. (2001)
10	14α-Hydroxywithanone	C_28_H_38_O_7_	19.56	486.26	487.25	469, 459, 451, 373, 343	Nittala and Lavie (1981)
11	Kaempferol	C_15_H_10_O_6_	19.65	286.05	287.32	269, 259, 258, 245, 203, 177, 175	Alam et al. (2011)
12	Withanolide O	C_28_H_36_O_5_	20.02	452.26	453.19	435, 417, 407, 263	Kirson et al. (1977)
13	Dihydrowithaferin A	C_28_H_40_O_6_	20.22	472.28	473.34	455, 437, 301, 285, 283	Girme et al. (2020)
14	Withanoside V	C_40_H_62_O_14_	20.47	766.41	767.15	723, 605, 587, 443, 425, 407, 253	Matsuda et al. (2001)
15	2,3-Dihydrowithanone-3beta-O-sulfate	C_28_H_40_O_10_S	20.75	568.23	569.24	551, 533, 515, 435, 417, 399, 361	Saleem et al. (2020)
16	Grossamide	C_36_H_36_N_2_O_8_	20.90	624.25	625.28	607, 488, 462, 338	Bolleddula et al. (2012)
17	Sominone	C_28_H_42_O_5_	21.07	458.30	459.34	441, 423, 405, 359	Iguchi et al. (2021)
18	Withanolide D	C_28_H_38_O_6_	21.74	470.27	471.18	435, 435, 425, 407, 371, 301	Gottlied and Kirson (1981)
19	Withaferin A	C_28_H_38_O_6_	22.01	470.27	471.10	453,441, 329, 301, 299, 281	Gottlied and Kirson (1981)
20	Withanolide G2	C_28_H_36_O_4_	23.21	436.26	437.31	419, 401, 373, 267, 265	Maurya (2017)
21	Withasomniferin A	C_28_H_38_O_5_	23.73	454.27	455.28	437, 427, 419, 409, 373, 355	Glotter et al. (1973)
22	Somniferine	C_36_H_36_N_2_O_7_	32.36	608.25	609.40	591, 580, 573, 549	Jamal et al. (1991)
23	3β-Ergosta-5,24-dien-3-ol	C_28_H_46_O	33.97	398.35	399.51	381, 338, 329, 315, 301, 217	Majumdar (1955)
24	3β-Stigmasta-5,24-dien-3-ol	C_29_H_48_O	35.80	412.37	413.58	395, 367, 353, 301, 237, 217	Majumdar (1955)
25	24,25-Dihydro-27-desoxywithaferin A	C_28_H_40_O_5_	36.51	456.29	457.61	439, 429, 413, 373, 331, 261	Lockley et al. (1974)
26	6,7-Benzochroman	C_13_H_12_O	42.10	184.09	185.10	167, 155, 141	Anjaneyulu and Rao (1997)

### 3.4 *In silico* and modeling studies

All the modeled structures of the LC-MS-annotated compounds were put through pharmacophore-based virtual screening using the PharmMapper platform (Wang et al., 2017) to determine how the *W. somnifera* extract exerts its anticancer activity. By mapping the major pharmacophore properties (i.e., the spatial arrangement of structural features) of a query structure, PharmMapper can search and recommend the most likely protein targets of this query structure. Similar protein targets are more likely to be bound by compounds that are structurally like those depicted in these pharmacophore maps. To determine which proteins might be targets of the metabolites indicated in the *W. somnifera* extract, we used the PharmMapper virtual screening platform. The retrieved results were ordered by their degree of conformity to the criteria (the Fit score). In this case, only cancer-related targets were chosen.

**FIGURE 5:**
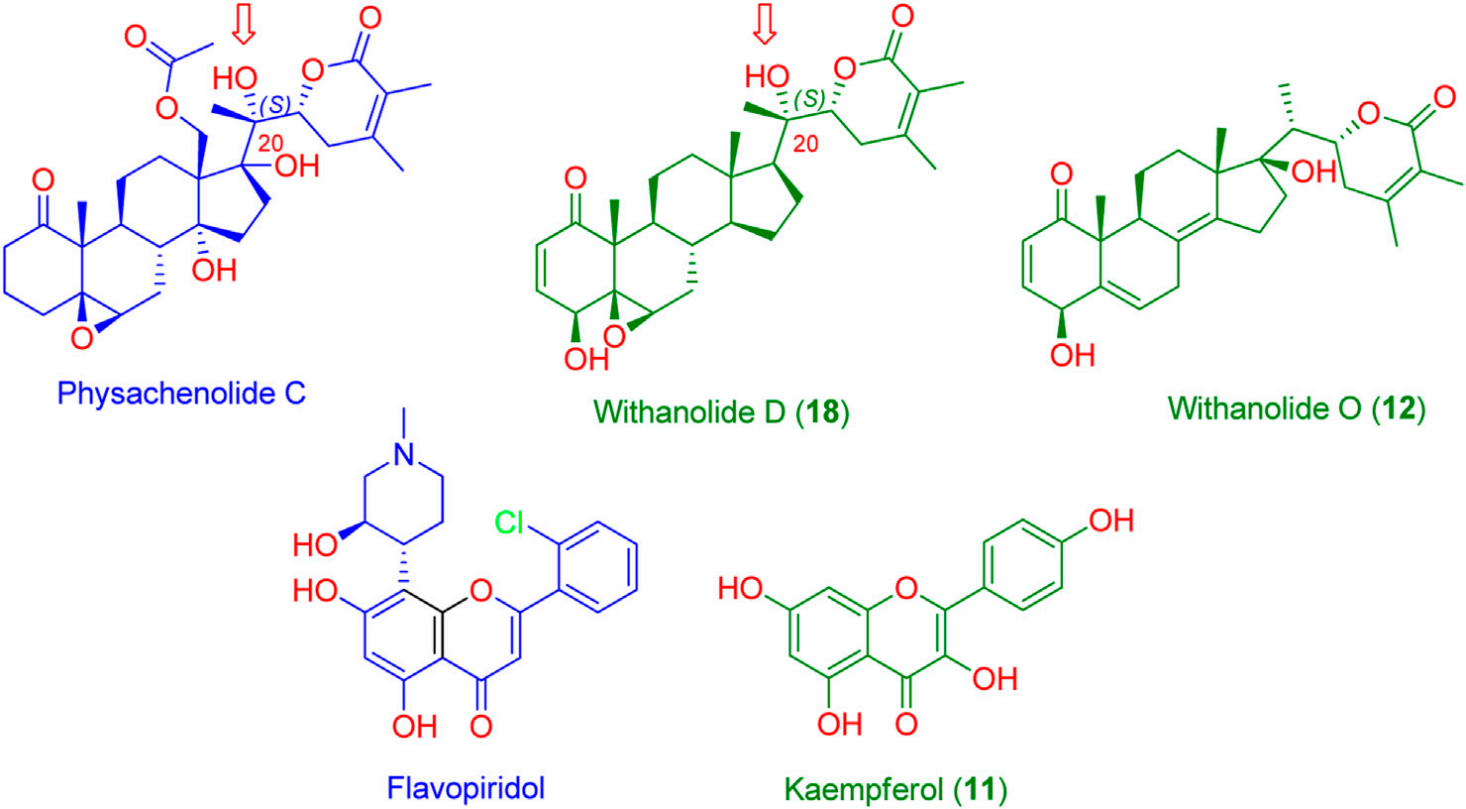
Structures of the probably anticancer metabolites (Green structures) according to PharmMapper-based virtual screening. Kaempferol (**11**) was predicted to be probably able to bind with CDK2 (PDB ID: 6GUB), while both withanolide D (**18**) and withanolide O (**12**) were found to be probably able to bind with BRD3 (PDB ID: 7S3B). Flavopiridol and physachenolide C (blue structures) are the reported co-crystalized inhibitors of both CDK2 and BRD3, respectively. Red arrows indicate the importance of the C-20 (S) hydroxyl group in stabilizing the binding of both withanolide D (**18**) and the co-crystalized inhibitor physachenolide C inside the BRD3 binding site.

As a result, CDK2 (PDB ID: 6GUB) was found to be among the top-scoring hits for kaempferol (**11**, Figure 5), and BRD3 (PDB ID: 7S3B) was found to be among the top-scoring hits for withanolides D (**18**) and O (**12**) (Figure 5) (Fit scores = 9.81, 11.12, and 11.67, respectively. Hence, these metabolites in *W. somnifera* extract can be considered the key bioactive compounds that may mediate its anticancer activity by inhibiting these proteins.

### 3.5 Molecular docking and dynamics simulation analyses

Modeled structures of the compounds were prepared and re-docked into the active sites of the proteins suggested as potential targets (CDK2 and BRD3). As a next step, we ran MD simulations for 100 ns on the obtained binding poses to verify the stability of the compounds’ binding within the active sites of the proposed protein targets.

**FIGURE 6:**
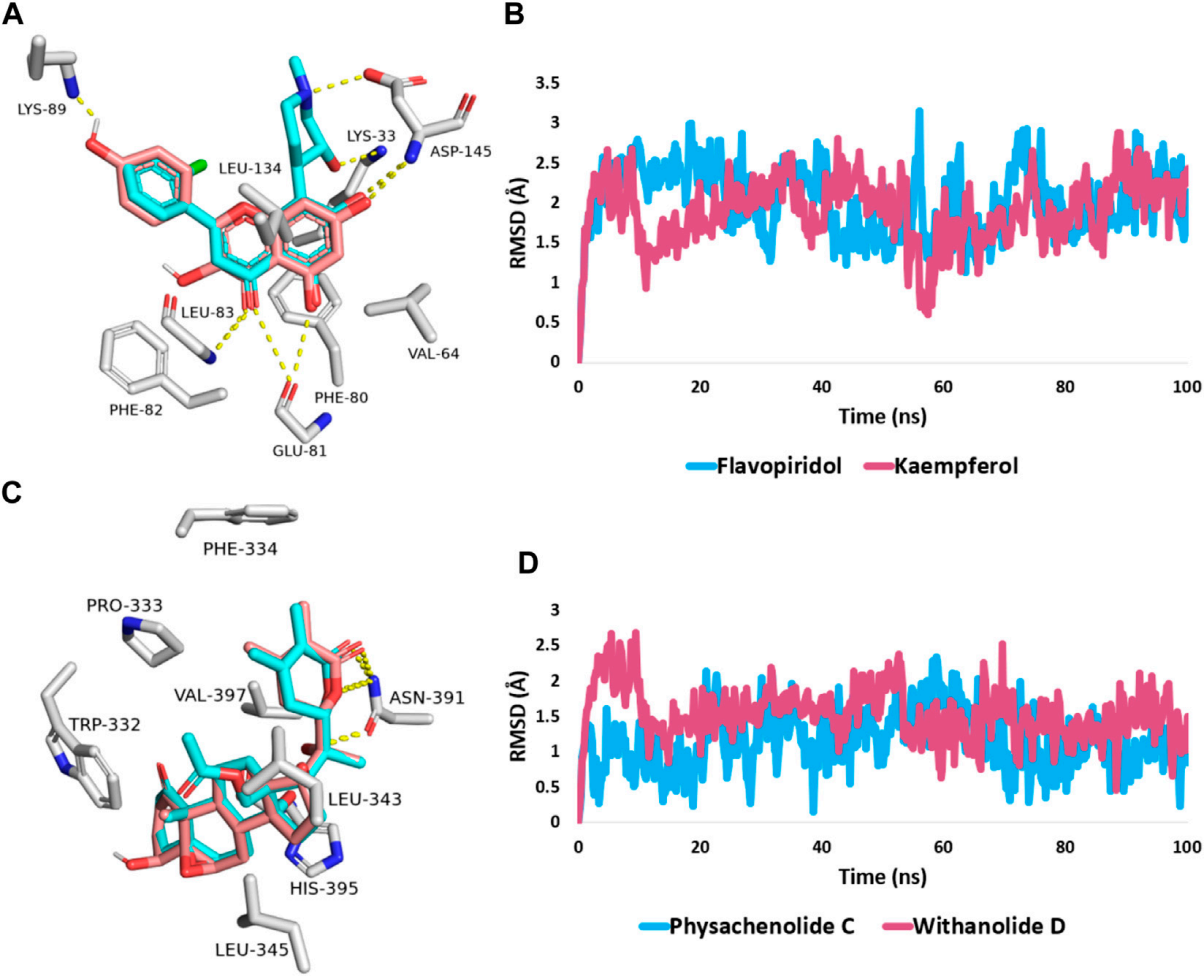
**(A,C)** Binding modes of kaempferol (**11**) and withanolide D (**18**) (brick red-coloured structure) in alignment with the co-crystalized inhibitors (Cyan-coloured structure) inside the binding site of CDK2 (PDB ID: 6GUB), and BRD3 (PDB ID: 7S3B), respectively. **(B,D)** RMSDs of both kaempferol (**11**) and withanolide D (**18**) inside the binding site of CDK2 and BRD3 throughout 100 ns-long MD simulation in comparison with the co-crystallized inhibitors.

First, docking the kaempferol **(11)** structure into the active site of CDK2 yielded binding modes and a docking score of −10.82 kcal/mol that was comparable to that of the co-crystallized inhibitor flavopiridol (docking score = −11.46 kcal/mol). Figure 6A shows that kaempferol **(11)** and the co-crystallized inhibitor flavopiridol were completely aligned with each other sharing most of the hydrophilic (i.e., H-bonding) and hydrophobic contacts (e.g., LYS-33, GLU-81, PHE-82, ASP-145, and VAL-64, PHE-80, LEU-134). Kaempferol **(11)** also exhibited more H-bonding with LYS-89.

Further, the 100 ns of MD simulation showed that the kaempferol **(11)** achieved a stable binding state, with an average RMSD of 2.18 Å, which is on par with the RMSD for the co-crystallized inhibitor flavopiridol (average RMSD = 2.07 Å; Figure 6B). Both retinol and QStatin were found to bind to the CDK2’s active site with a high degree of stability, as evidenced by their calculated absolute binding free energies (ΔGBind) within the CDK2’s binding site (ΔGBind = −9.47 and −9.89 kcal/mol, respectively). Previous modelling and MD simulation results suggest that kaempferol **(11)** can putatively target CDK2 exerting possible anticancer activity. On the other hand, the docking scores for withanolide D (18) and withanolide O (**12**) alongside the co-crystallized ligand, physachinoloide C three structures inside BRD3’s active site were −8.24, −8.67, and −8.39 kcal/mol, respectively.

The binding mechanism for both withanolide D **(18)** and withanolide O **(12)** was in good alignment with the co-crystallized ligand, physachinoloide C. The three molecules were able to form two H-bonds with ASN-391 via their lactone rings, however, only withanolide D **(18)** alongside the co-crystallized inhibitor was able to establish an additional H-bond with ASN-391 via their C-20 hydroxyl group (Figure 6C).

**FIGURE 7:**
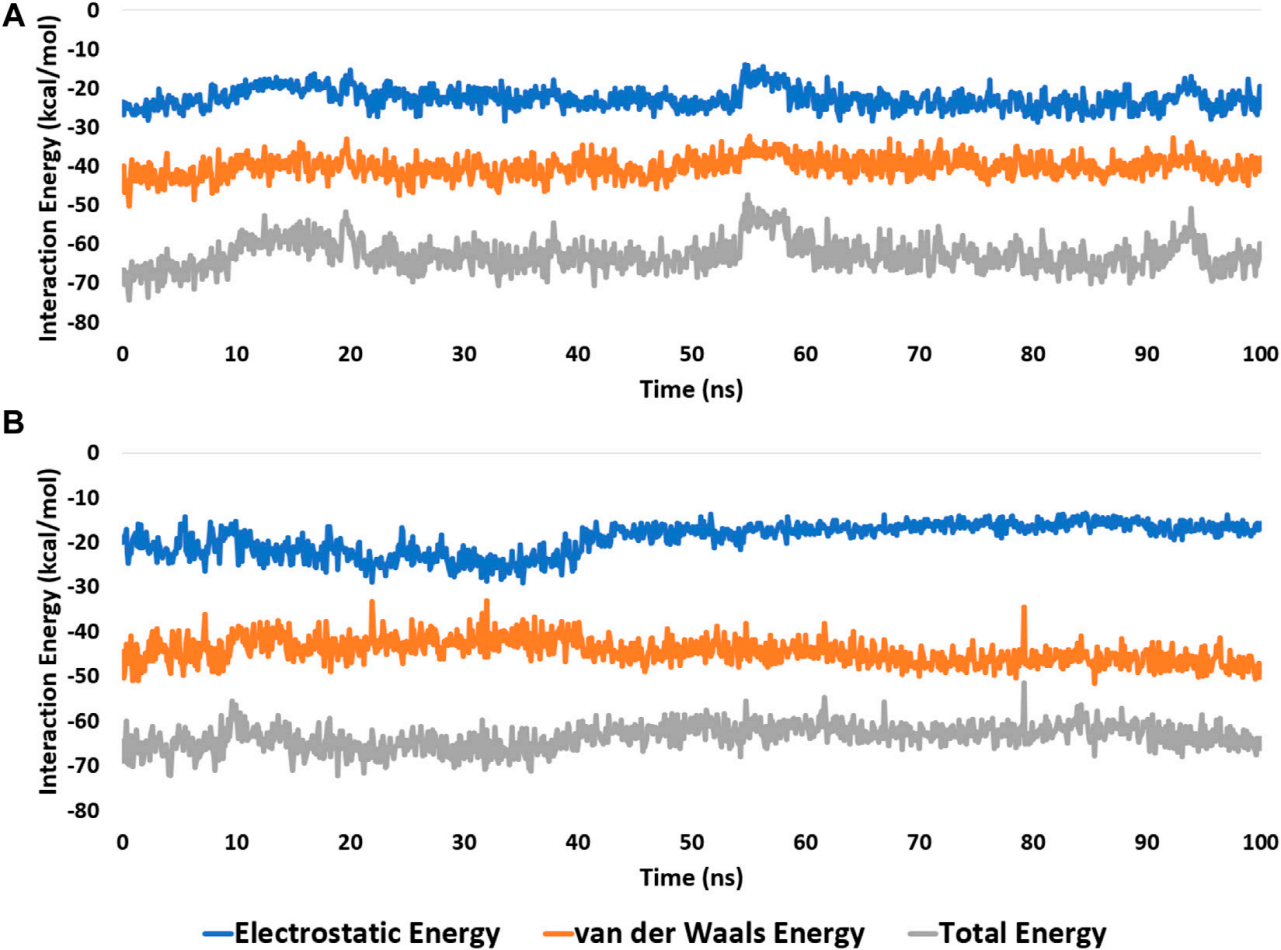
Interaction energies (i.e., electrostatic and van der Waals interaction energies) of kaempferol (**11**) and withanolide D (**18**) inside the binding site of CDK2 (PDB ID: 6GUB), and BRD3 (PDB ID: 7S3B), respectively. **(A,B)**, respectively over the course of 100 ns-long MD simulation.

**FIGURE 8:**
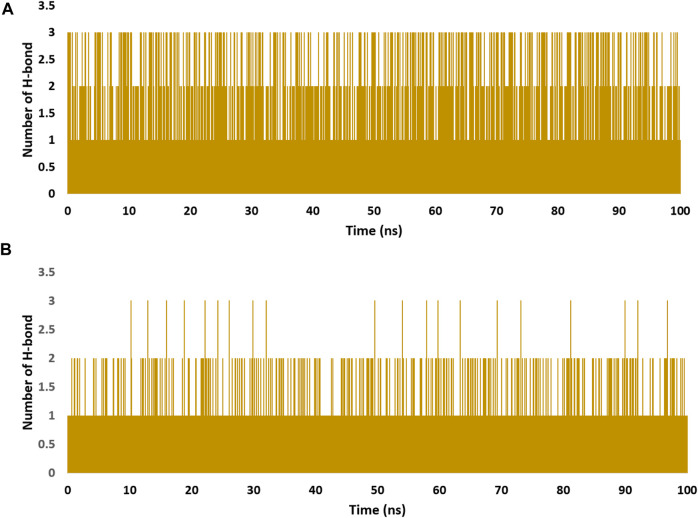
Number of H-bonds detected for of kaempferol (**11**) and withanolide D (**18**) inside the binding site of CDK2 (PDB ID: 6GUB), and BRD3 (PDB ID: 7S3B), respectively **(A,B)**, respectively over the course of 100 ns-long MD simulation. The cut-off distance for H-bonds was set to be 2.5 Å.

These findings were further supported by 100 ns MD simulations of the docking poses (Figure 6D), which revealed stable binding of withanolide D (18) within BRD3’s active site, with an average RMSD value convergent to that of the co-crystallized inhibitor (1.67 Å and 1.55 Å, respectively). Withanolide O (**12**), on the other hand, did not achieve stable binding like its close derivative withanolide D, and it left the BRD’s active site completely after 48 ns of MD simulation. This observation indicates that the H-bond between the C-20 hydroxyl group in both withanolide D (**18**) and the co-crystallized inhibitor, physachinoloide C, and ASN-391 is essential for the binding stability of such scaffolds. The calculated ΔGBind of withanolide D **(18)** within the BRD3’s binding site is almost identical to that of the co-crystallized ligand (−9.12 and −9.19 kcal/mol, respectively) indicating that withanolide D **(18)** is likely act as BRD3 inhibitor just like the co-crystallized inhibitor, physachinoloide C. Consequently, the overall interaction energies of both compounds (i.e., kaempferol and withanolide D) averaged around −62.45 and −68.13 kcal/mol, respectively (Figure 7). Furthermore, both compounds established stable hydrophilic contacts, particularly H-bonds, which were found to be between 1 and 3 H-bonds for kaempferol (**11**) and 1 to 2 H-bonds for withanolide D (**18**) throughout the course of simulation (Figure 8).

## 4 Discussion

Oral cavity and oropharyngeal cancer frequencies have increased during the past 20 years at an alarming rate (Shield et al., 2017). The discovery of chemotherapeutic/chemoprophylactic anticancer drugs from plant-based edible natural sources is attracting researchers’ attention due to their potential tolerance, low toxicity, and probable *in situ* triggering cascade of anticancer process. *W. somnifera* herbal supplement (used as a drink) is an effective adaptogen and anti-stress agent that improves physical and mental performance, optimizes attention, and promotes restful sleep (Speers et al., 2021). Moreover, its phytoconstituents have been intensively studied as immunomodulatory and anticancer agents against numerous cancer cell lines from almost all body areas, however, its potential role against oral cancers has received scant attention in the literature (Kashyap et al., 2022). Therefore, hydroalcoholic root and fruit *W. somnifera* extracts and their solvent fractions were screened against 4 oral tumors (Ca9-22, HSC-2, HSC-3, and HSC-4), and 3 normal oral (HGF, HPLF, and HPC) cells, in comparison with the positive standard anticancer drugs, such as doxorubicin, 5-FU, and melphalan, followed by flow cytometric analysis to annotate the possible cytotoxicity mechanism. The active cytotoxic principles in the root EtOAc (W-R EtOAc) and butanol (W-R BuOH) fractions exhibited noticeable selective cytotoxicity against the different cancer cells. Stronger cytotoxicity of these fractions was observed against the Ca9-22 cell line (CC_50_ = 51.8 and 40.1 μg/mL, respectively), which is comparable to the effects of the 5-FU (CC_50_ = 69.4 μM) and melphalan (CC_50_ = 36.3 μM) on the same cancer cell line (Table 1). The selectivity of the drugs towards tumours rather than normal tissues is a vital quality for ensuring the treatment of cancer patients with minimum side effects. Therefore, the active fraction tumor-specificity was calculated (TS = 2.3 and 5, respectively) (see result section), which is comparable to that of 5-FU and melphalan (TS = 2.6 and 10.1, respectively). Also, the calculated potency-selectivity expression (see *results* section) (PSE = 2.9 and 6.6, respectively) was much higher than that of 5-FU (PSE = 0.7). The relative sensitivity of the gingival Ca9-22 and HGF cells (TS = 3.6 and 7.3, and PSE = 6.9 and 18.3 were also comparable with that of 5-FU and melphalan (TS = 14.4 and 5.0 and PSE = 20.8 and 13.7, respectively) (Table 1).

To highlight the possible cytotoxic mechanism of these selectively potent W-R EtOAc and W-R BuOH fractions, flow cytometric analysis was conducted on the most sensitive Ca9-22 cells. The result revealed that Act. D and W-R EtOAc (125 μg/mL) suppress cell growth and induce subG_1_ accumulation, suggesting a link between apoptosis and cytotoxicity as previously reported (Ma et al., 2021). Also, a marked suppression of cell growth in the G_1_ phase was observed in both EtOAc (125 μM) and BuOH (62.5, 125, and 250 μM) treated cells. However, only EtOAc (125 μM) seems to inhibit DNA synthesis during the S phase. These changes consequently lead to the growth arrest in the populations of the G_2_ or M phase in Act. D, W-R EtOAc (125 μM), and W-R BuOH (250 μM) treated oral cancer cells. Previous studies reported the ability of *W. somnifera’s* main active component, withaferin A (**19**), to induce apoptosis in the cancer cells Ca9–22 by arresting the G_2_/M cell cycle through the generation of ROS and mitochondrial polarization suggesting the occurrence of oxidative stress-facilitated killing of oral cancer cells (Das et al., 2017; Peng et al., 2021). G_1_ phase arrest can consequently lead to either G_1_ arrest pending repair, DNA repair, and return to the cell cycle or elimination via apoptosis (Kaina, 2003). These results agreed with previous research, where withanolide C (another active component) inhibited the proliferation of breast cancer cells through DNA damage and oxidative stress-mediated apoptosis (Yu et al., 2020).

The UHPLC-Q-TOF-MS/MS analysis enabled the tentative identification of twenty-six components, mainly withanolides, and withanosides along with some alkaloids, flavonoids, and steroids (Table 2), which are typical of *W. somnifera* metabolites.

Several *in vitro* studies confirmed the anticancer role of several members of these *W. somnifera* metabolites. Flavonoids, including daidzein **(3)**, genistein **(4)**, and kaempferol **(11)** have proven activities against various cancer cell lines. Daidzein **(3)** has anti-cancer activity against prostate LNCaP (lymph node carcinoma of the prostate), androgen-independent prostate carcinoma cell line (DU145), and prostatic adenocarcinoma (PC3) cell lines (Adjakly et al., 2013; Ranjithkumar et al., 2021). Genistein **(4)** acts through different pathways as a PTK (protein tyrosine kinase) inhibitor against breast cancer cells (Peterson and Barnes, 1996) and causes upregulation of E-Cadherin in MOLT4 (human acute T lymphoblastic leukaemia), JURKAT (human immortalized T cell leukemia), and ALL (acute lymphoblastic leukemia) cell lines (Namordizadeh, 2019). Moreover, it decreased VEGF (vascular endothelial growth factor), HIF-1α (hypoxia inducible factor 1 subunit alpha), and NF-κB (nuclear factor kappa B), and increased the p21 tumor suppressor in the breast cancer cell line (Mukund, 2020). Moreover, it improved the repressive effect of GCP (genistein combined polysaccharide) on the proliferation and apoptosis of androgen-sensitive LNCaP cells (XU et al., 2020). Kaempferol **(11)** causes apoptosis and arrests the murine melanoma B16 cells (Qiang et al., 2021) and acts through different pathways such as epidermal growth factor receptor/mitogen-activated protein kinas/a serine/threonine protein kinase (EGFR/MAPK/AKT) pathways in human cervical cells (Tu et al., 2016) and blocked EGFR-related pathways in pancreatic cancer (Lee and Kim, 2016). Moreover, withanolides, a group of ergostane-based C28 steroidal lactones with wide structural heterogeneity due to polyoxygenation at distinct locations of the skeleton, showed various cytotoxicity mechanisms against cancer cell lines. Withaferin A (**19**), the main withanolide in *W. somnifera*, modulated TGF-β (Transforming growth factor-β) signaling in endometrial cancer (Xu et al., 2021) and suppressed STAT3 (signal transducer and activator of transcription 3) in multiple myeloma and neuroblastoma (Yco et al., 2014). In addition, various molecular mechanisms involved in the antiproliferative effects, as well as comprehensive details about its *in vitro*, *in vivo*, and *in silico* manners as anticancer agent are gathered in a recent review article (Sivasankarapillai et al., 2020).

Herein, a comprehensive *in silico*-based investigation illustrated the flavonoid and withanolide contents of *W. somnifera* extract having a key role in its anticancer activity against OSCC. According to the preliminary pharmacophore-based screening, kaempferol **(11)**, and withanolides D **(18)** and O **(12)** were suggested to bind with both CDK2 and BRD3, respectively, as cancer-relevant target proteins. Cell cycle progression depends on the activity of CDKs, which are the catalytic subunits of a broad family of heterodimeric serine/threonine protein kinases (Krithiga and Jayachitra, 2012). Bromodomain-containing protein 3 (BRD3), encoded by the BRD3 gene in humans belongs to the Bromodomain and Extra-Terminal motif (BET) protein family. These proteins associate with acetylated lysine residues on histones and transcription factors (Lai et al., 2021). Depletion of BRD3 has been correlated to slower growth in prostate and medulloblastoma cancer models (Belkina and Denis, 2012).

Targeted degradation of BRD proteins (BRD2, BRD3, and BRD4) has been linked to a better prognosis in various AML cell lines (Zhang et al., 2022). Moreover, several earlier studies have shown a substantial relationship between CDK2 expression and oral squamous cell cancer progression (Mihara et al., 2001; Shintani et al., 2002; Huang et al., 2018).

Therefore, molecular docking of the identified compounds with these oncogenic proteins was conducted, and their binding properties were discovered by molecular dynamics (MD) simulation. The MD simulation study and the ΔG Bind calculation showed that both kaempferol (**11**) and withanolide D **(18)** but not its close derivative withanolide O **(12)** are possible inhibitors of CDK2 and BRD3, respectively. These findings also highlighted a key structure requirement (i.e., the presence of a hydroxyl group at the C-20) of the withanolides to achieve stable binding with BRD3.

Kaempferol **(11)** inhibited cancer cell growth and proliferation by suppressing the cyclin-dependent kinases (CDKs) activity and the associated cyclins involved in the regulation of the cell cycle transition from G_1_ to S phase (Choi and Ahn, 2008; Qattan et al., 2022). Kaempferol **(11)** reduced CDK2 and CDK4 protein levels, as well as those of cyclin A, cyclin D1, and cyclin E in a dose-dependent manner (Choi and Ahn, 2008). It also induced autophagy through adenosine monophosphate-activated protein kinase (AMPK) and protein kinase B (PKP) signaling molecules and caused G_2_/M arrest via downregulation of CDK1/cyclin B in the human hepatic cancer cell line SK-HEP-1. Furthermore, through both intrinsic and extrinsic apoptotic pathways, it may play a part in triggering apoptosis in a variety of cancer cell types, which may have an anti-cancer effect. In addition, kaempferol can target many molecular-signaling pathways such as vascular endothelial growth factor (VEGF), signal transducer and activator of transcription (STAT), p53 (a tumor suppressor protein), PI3K-PKP (phosphoinositide-3-kinase–protein kinase B), NF-κB (Nuclear factor kappa-light-chain-enhancer of activated B cells), and ERRα (estrogen-related receptor α) signaling pathways, depending on what cancer cell lines were used. It is important to generalize what biochemical reactions happen after the binding of kaempferol to the targets (Amjad et al., 2022). It was shown that CDK2 controls the G_1_/S transition and the transcription factor E2F’s activity, which are necessary for DNA replication and repair (Satyanarayana and Kaldis, 2009; Li et al., 2022).

Previous study on synthetic pyrazolo [1,5-a]pyrimidine derivatives {2-[1-Cyano-2-(4-methoxyphenyl)vinyl]-5,7-diphenylpyrazolo [1,5-a]-pyramidine-3-carbonitrile (A) and 2-[1-Cyano-2-(4-methoxyphenyl)vinyl]-5,7-diphenylpyrazolo [1,5-a]-pyramidine-3-carbonitrile (B)} demonstrated strong inhibitory activity against CDK-2, and both compounds (A and B) arrested the cell cycle at G_2_/M phase in the cell cycle assay using the HepG-2 cancer cell line (Metwally et al., 2021; Mohammed and Elmasry, 2022). Similarly, taxol inhibition of the CDK2 led to cell cycle arrest at G_2_/M phase (Sang et al., 2022).

These literature data are consistent with the observed growth arrest in the Ca9-22 cancer cell populations in the G_2_ or M phase by W-R EtOAc (125 μM) and W-R BuOH (250 μM) and highlight the involvement of CDK2 inhibition of as a molecular anticancer mechanism.

Anticancer activity of *W. somnifera*’s withanolides, including those identified in the herein mass analysis, has been studied widely. Some research reported withanolide D **(18)** antiproliferative effect on multiple myeloma cells, and exhibited a cytostatic effect in both drug-resistant and drug-sensitive multiple myeloma cells by inducing cell death and apoptosis in a dose- and time-dependent manner (Issa et al., 2017). Withanolide D **(18)** has been shown to induce apoptosis in leukaemia by activating the neutral sphingomyelinase 2 (nSMase2) enzyme and modulating the phosphorylation of the JNK and p38MAPK pathways (Mondal et al., 2010). In a study by Wang et al. (2017), it was discovered that withanolide D inhibits the proliferation of cancer cells by lowering the expression of the BRD3 protein (Cheung et al., 2021). The BRD3 inhibition in lung cancer cell lines has been shown to induce G_2_/M cell cycle arrest and increased apoptosis rate (Yoo et al., 2020).

These findings suggest the potential of W-R EtOAc and W-R BuOH fractions to induce G_2_/M phase accumulation through CDK2 and BRD3 inhibition. Kaempferol **(11)** and withanolide D **(18)** are likely the active anti-oral cancer metabolites in *W. somnifera* extract. However, further research on purified compounds from the W-R EtOAc and W-R BuOH fractions is demanded to confirm this effect and understand the predominant transition of cells to the subG_1_ population instead of G_1_ and S phases is needed (Neganova et al., 2011).

Despite the importance of determining the type of cell death, in our preceding report we have detected that SN-38, an *in vivo* active antitumor metabolite of the antitumor drug irinotecan, could induce diverse types of cell death in two oral squamous cell carcinoma cell lines: (ⅰ) apoptosis mediated by caspase-3 activation as well as fragmentation of the internucleosomal DNA in HSC-2 cell type. (ⅱ) the formation of autophagosome and secondary lysosome (autophagy) in the HSC-4 cell type. Re-treatment with autophagy inhibitors (3-methyladenine, bafilomycin A1) and caspase inhibitor (Z-VAD-FMK) dramatically reduced the cell death of HSC-2 and HSC-4 cells, respectively (Tamura et al., 2012). This implies that tumor-specificity indicators (TS and PSE) are more important for the future administration of chemotherapy for OSCC than the kind of cell death. We have recently reported that two 3-styrylchromone derivatives, 7-methoxy-3-[(1*E*)-2-phenylethenyl]-4*H*-1-benzopyran-4-one (Compound A) and 3-[(1*E*)-2-(4-hydroxyphenyl) ethenyl]-7-methoxy-4*H*-1-benzopyran-4-one (Compound B), showed comparable TS values against human OSCC cell lines with 5-FU, cisplatin and doxorubicin. Quantitative structure-activity relationship (QSAR) prediction based on the Tox21 (21st century toxicology program) database suggested that compounds A and B may inhibit the signalling pathway of estrogen-related receptor α (ERRα), but not the other 58 signalling pathways (Abe et al., 2023). Thus, it is urgent to test the possibility that selective inhibition of ERRα may also be involved in the anticancer activity of kaempferol (**11**) and withanolide D (**18**), and related compounds.

## 5 Conclusion

*W. somnifera* root EtOAc portion of the hydromethanolic extract exerted superior cytotoxic activity against OSCC. This was confirmed through the *in vitro* investigation of various extracts and fractions against Ca9-22, HSC-2, HSC-3, and HSC-4 oral cancer cell lines. A study using flow cytometry revealed morphological alterations and subG_1_ accumulation in response to W-R EtOAc (125 μg/mL) on the Ca9-22 cell line, indicating apoptotic cell death as the mechanism of cytotoxicity. The two-stage mass spectroscopic analysis highlighted 26 compounds typical of the *W. somnifera* phytoconstituents. *In silico*-based investigation of the identified compounds discovered that kaempferol **(11)** and withanolide D **(18)** have a key role in its anticancer activity and are possible inhibitors of the oncogenic proteins CDK2 and BRD3, respectively. However, since CDK2 and BRD3 are also involved in the transition to the G_1_/S phase of the cell cycle (Neganova et al., 2011), many other CDK series may be involved in the cytotoxicity mechanism of the W-R EtOAc and W-R BuOH fractions, which require further assay studies. Despite the lack of *in vivo* investigation, which is a drawback of this study, our obtained results extend the exploitation of *W. somnifera’s* anticancer potential to involve oral malignancies. The two most likely active anti-oral cancer metabolites in *W. somnifera* extract, kaempferol (**11**) and withanolide D (**18**), are worth additional *in vitro* and *in vivo* research in future work.

## Data Availability

The original contributions presented in the study are included in the article/Supplementary material, further inquiries can be directed to the corresponding author.
